# Understanding of Coupled Terrestrial Carbon, Nitrogen and Water Dynamics—An Overview

**DOI:** 10.3390/s91108624

**Published:** 2009-10-29

**Authors:** Baozhang Chen, Nicholas C. Coops

**Affiliations:** 1 LREIS Institute of Geographic Sciences & Nature Resources Research, Chinese Academy of Sciences, Beijing 100101, China; 2 Department of Forest Resources Management, Faculty of Forestry, University of British Columbia 2424 Main Mall, Vancouver, BC V6T 1Z4, Canada; E-Mail: coop.nicholas@ubc.ca

**Keywords:** terrestrial carbon and water dynamics, ecohydrological modeling, remote sensing, eddy-covariance flux tower, scaling

## Abstract

Coupled terrestrial carbon (C), nitrogen (N) and hydrological processes play a crucial role in the climate system, providing both positive and negative feedbacks to climate change. In this review we summarize published research results to gain an increased understanding of the dynamics between vegetation and atmosphere processes. A variety of methods, including monitoring (e.g., eddy covariance flux tower, remote sensing, etc.) and modeling (i.e., ecosystem, hydrology and atmospheric inversion modeling) the terrestrial carbon and water budgeting, are evaluated and compared. We highlight two major research areas where additional research could be focused: (i) Conceptually, the hydrological and biogeochemical processes are closely linked, however, the coupling processes between terrestrial C, N and hydrological processes are far from well understood; and (ii) there are significant uncertainties in estimates of the components of the C balance, especially at landscape and regional scales. To address these two questions, a synthetic research framework is needed which includes both bottom-up and top-down approaches integrating scalable (footprint and ecosystem) models and a spatially nested hierarchy of observations which include multispectral remote sensing, inventories, existing regional clusters of eddy-covariance flux towers and CO_2_ mixing ratio towers and chambers.

## 1. Introduction

The terrestrial biosphere plays a crucial role in the climate system providing both positive and negative feedbacks to climate change [1]. The terrestrial carbon (C) cycle is closely linked to hydrological and nutrient controls on vegetation [2-3]. Understanding the coupled terrestrial C, nitrogen (N) and water cycle is required to gain a comprehensive understanding of the role that terrestrial ecosystems play in the global climate change. Much progress has been made in gaining insight of the coupling processes between C, N and water cycles across a range of time and spatial scales [4-9]. Since the early 1990s, there has been an increased interest in monitoring of the CO_2_, water vapor and energy exchange between the atmosphere and terrestrial ecosystems by a variety of methods, such as the eddy-covariance techniques (EC), satellite and other airborne remote sensing, CO_2_ concentration and isotope measurements. Meanwhile, there are various kinds of models have been developed to better understanding of these processes and for large-scale C and water budgeting.

The large number of papers published since the 1980s on the terrestrial and C and water cycles have resulted in the publication of several major reviews from different perspectives. For example, Running *et al.* [10] described a blueprint for more comprehensive coordination of the various flux measurement and modeling activities into a global terrestrial monitoring network by reviewing the literature published before the middle of 1990s. Baldocchi [9] recently provided a comprehensive review of research results associated with a global network of C flux measurement systems. The topics discussed by this review include history of the network, errors and issues related with the EC method, and a synopsis of how these data are being used by ecosystem and climate modellers and the remote-sensing community [9]. Kalma *et al.* [11] reviewed satellite-based algorithms for estimating evepotranspiration (ET) and land surface temperatures at local, regional and continental scales, with particular emphasis on studies published since the early 1990s; while Verstraeten *et al.* [12] provided a comprehensive review of remote sensing methods for assessing ET and soil moisture content across different scales based on the literature published after 1990s. Marquis and Tans [13] reviewed satellite-based instruments on CO_2_ concentration measurements.

In this review article, we distil and synthesise the rapidly growing literature on C and water cycles across local to global spatial scales and over a range of time scales. To give the reader a perspective of the growth of this literature, a search of Web of Science produced over 2000 papers with the key words ‘ecosystem carbon, water and nitrogen cycles’ published since 1990 which is indicative of the large amount of research recently being undertaken on these topics. In order to filter through this large body of literature, we concentrated on papers discussing on the coupling processes between C, water and N cycles and we extracted information from a database of published results that we have collated during the past decade (available on request). In terms of content, the report covers the state of knowledge, monitoring and modeling of the coupled terrestrial C and water cycles. Our aim is to highlight the recent advances in this field, and propose areas of future research based on perceived current gaps in the literature.

The review is divided into several inter-connected sections. First, we review the scientific background of the linkage between terrestrial ecosystems and climate, and revise the state of knowledge on terrestrial C cycling, coupling of the C and water cycles, and coupling of the C and N cycles. Second, we discuss the ground-based and satellite-based monitoring methods and observation networks associated with measuring C and water fluxes, CO_2_ concentration and C isotopes. Third, we report on the recent advances in modeling approaches associated with the terrestrial biochemical and hydrological studies. Fourth, we discuss research gaps in C sinks/sources estimates and finally, we discuss the current research trends and the near-future directions in this field and propose an upscaling framework for landscape and regional C and water fluxes estimates.

## 2. Scientific Background and State of Knowledge

### 2.1. Overview of Terrestrial Ecosystems and Climate

**Figure 1.**
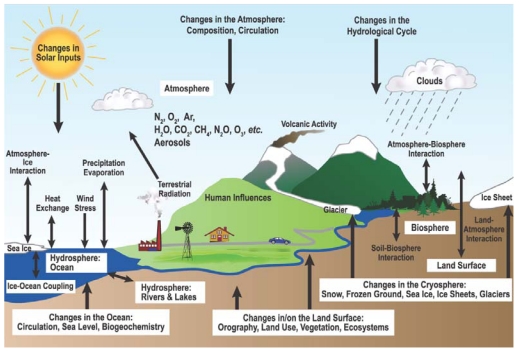
Schematic view of the components of the climate system, their processes and interactions [1].

The climate system is controlled by a number of complex coupled physical, chemical and biological processes (Figure 1). The terrestrial biosphere plays a crucial role in the climate system, providing both positive and negative feedbacks to climate change through biogeophysical and biogeochemical processes [1]. Couplings between the climate system and biogeochemistry are mainly through tightly linked dynamics of C and water cycles. The importance of coupled C and water dynamics for the climate system has been increasingly recognized [2-8]; however the mechanisms behind these coupled cycles are still far from well understood.

### 2.2. Terrestrial C Cycling

**Figure 2.**
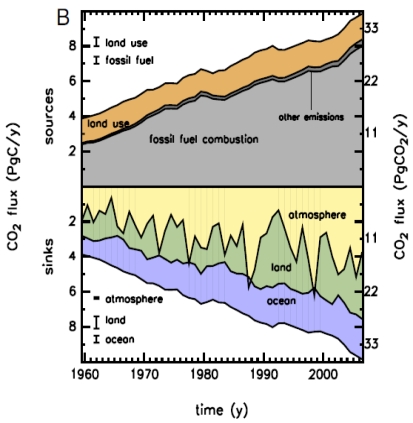
Global CO_2_ budget from 1959 to 2006. Upper panel: CO_2_ emissions to the atmosphere (sources) as the sum of fossil fuel combustion, land-use change, and other emissions. Lower panel: The fate of the emitted CO_2_, including the increase in atmospheric CO_2_ plus the sinks of CO_2_ on land and in the ocean [14].

One of the crucial issues in the prognosis of future climate change is the global budget of atmospheric CO_2_. The growth rate of atmospheric CO_2_ is increasing rapidly. Three processes contribute to this rapid increase: fossil fuel emission, land use change (deforestation), and ocean and terrestrial uptake. As shown in Figure 2, terrestrial C budgets have large uncertainties and interannual variability.

Terrestrial ecosystems mediate a large part of CO_2_ flux between the Earth's surface and the atmosphere, with ∼120 Pg C yr^-1^ taken up by photosynthesis and roughly the same amount released back to the atmosphere by respiration annually [1,15]. Imbalances between gross ecosystem photosynthesis or gross primary productivity (GPP) and ecosystem respiration (*R_e_*) lead to land surfaces being either CO_2_ sinks or sources. C sequestration by global terrestrial ecosystems is estimated to be about 1-2 Gt C yr^-1^ [1,15]. A detailed understanding of the interactive relationships in atmosphere-biosphere exchange is relevant to ecosystem-scale analysis and is needed to improve our knowledge of the global C cycle [16]. The metabolism of terrestrial ecosystems is complex and highly dynamic because ecosystems consist of coupled, non-linear processes that possess many positive and negative feedbacks [17,18]. Complex features of ecosystem metabolism are relatively unknown and how C budget of major ecosystems will respond to changes in climate is not quantitatively well understood [19-23].

### 2.3. Coupling of the C and Water Cycles

Thermodynamically, a terrestrial ecosystem is an open system. Therefore, hydrological and C cycles are closely coupled at various temporal and spatial scales [7,24-30]. C uptake for example, is closely coupled to water loss by ecosystems mainly through leaf stomatal pathway governed principally through leaf conductance [31-33]. Soil organic C decomposition is very sensitive to soil moisture content via microbial activity and other processes [8,25,30,34,35]. The flux of terrestrial organic C by river runoff to the ocean and wetland discharge is an important component of the global organic C cycle [36,37]. It is estimated that 0.25 × 10^15^ g dissolved organic carbon (DOC) is discharged to the ocean by the world rivers each year [38]. The land surface hydrological processes (in particular the terrestrial river systems) play an important role in transport of dissolved and particulate organic C from terrestrial to marine ecosystems [37]. However, the interactions between C and water cycles and the mechanisms how these interactions will shape future climatic and biosphere conditions are far from well understood.

### 2.4. Coupling of the C and N Cycles

N is an essential element that can limit the growth of living organisms across a wide range of ecosystems [39,40]. However, global inputs to the terrestrial N cycle have doubled in the past century due to anthropogenic activities, particularly fertilizer use and fossil fuel burning [41,42]. High atmospheric N and N depositions have changed the dynamics of N and C in many ecosystems of developed countries (e.g., in Europe and North America) [43], with the impact in other Asian developing countries projected to increase over the next few decades [44]. N addition to forest and other ecosystems will affect the health and vitality of ecosystems and the terrestrial C cycle [45,46]. There is a continuing discussion on whether N deposition has positive (e.g., increases in forest growth and C sequestration) or negative (e.g., increases in nitrate leaching, reduction in forest growth and C sequestration) effects on an ecosystem depends on the N status of the system and the rate and duration of N deposition [45,46]. Bauer *et al.* [47] summarized that (1) if the remaining available N does not exceed the capacity for N uptake by vegetation net primary productivity (NPP), C sequestration may be enhanced; (2) if deposition rates exceed the capacity for N uptake, nutrient imbalances can lead to forest decline due to N saturation [48,49]. Aber *et al.* [45,46] hypothesized that forest ecosystems positively respond to N addition in the short-term, while negatively respond to N addition over the long-term. N fertilization in northern temperate zones has been estimated to enhance C storage by 0.3–0.5 Pg C per year [50] with Pregitzer *et al.* [51] reporting that simulated chronic N deposition has increased C storage in northern temperate forests. Olsson *et al.* [52] found that fertilization of a boreal Norway spruce stand led to a three-fold increase in aboveground productivity, possibly due to decreased C allocation to roots in response to higher nutrient availability. Leggett and Kelting [53] found that N fertilization of Loblolly pine plantations not only increased aboveground and belowground biomass but also increased soil C pools. However, other estimates suggest that N loading on ecosystems only makes a minor contribution to C sequestration [54] or does not likely account for significant C storage [55,56] or in some cases may actually reduce ecosystem productivity and C storage [46,57].

N fertilisation may affect the pool of soil organic carbon (SOC) through the changes of litterfall. Both negative [58] and positive [59] effects of N fertilisation on root biomass have been observed. In a detailed review Nadelhoffer [60] found it is likely that N deposition will decrease fine-root biomass, however, stimulate fine-root turnover and production. N addition/fertilization to soils has also shown variable effects on SOC storage by enhanced, reduced or unchanged heterotrophic and/or autotrophic respiration as a response to litter C/N ratio [61-68]. With increasing rates of anthropogenic N deposition [41,42], there is a strong need to understand links between ecosystem N inputs and C sequestration.

## 3. Monitoring of C and Water Cycling in Terrestrial Ecosystems

### 3.1. Global Flux Tower Network (FLUXNET)

The EC technique is commonly used to directly measure the CO_2_, water vapor and energy exchange between the atmosphere and terrestrial ecosystems [9]. A global network of collaborating regional networks, FLUXNET, began in 1997 [9] and today, there exist more than 400 EC-flux towers across the globe. EC measurements are a rich source of information on temporal variability and environmental controls of CO_2_ exchange between the atmosphere and terrestrial ecosystems [69]. These global EC datasets allow us to (1) explore emergent-scale properties by quantifying how the metabolism of complex ecosystems respond to perturbations in climate variables on diurnal, seasonal, interannual and decadal time scales and elucidate physical and biological controlling factors [9,69]; (2) examine carry-over effects that may be introduced by either favorable or deleterious conditions during antecedent years [70]; (3) observe a disturbance and the recovery from it or to span a natural sequence of ecological development coupled with fluctuations in climate [71,72]; and (4) test and validate ecosystem process models [73,74], since most of these models span timescales from hours to decades. Although the available EC data have been rapidly accumulating, there are some issues associated with its use due to difficulties/uncertainties in (i) assessing/interpreting the associated measuring biases of EC data, and (ii) upscaling of the EC fluxes at the ecosystem (typically less than 1–3 km^2^ for each site) to larger scales, e.g., landscape and regional scales.

### 3.2. CO_2_ Concentration Measurements, Data Assimilation and CarbonTracker

Observations of CO_2_ over the continent within the atmospheric boundary layer reflect exchange processes occurring at the surface at a regional scale (10^2^–10^5^ km^2^). The flux information contained in CO_2_ concentration data represents footprints of up to 10^5^ km^2^ [75-77], which is several orders of magnitude larger than the direct EC-flux footprint. These measurements form a record of integrated net CO_2_ exchange from multiple processes, geographic areas, and times [78]. CO_2_ concentration measurements are made from tall towers [79-81] or balloons that reach the top of the planetary boundary layer (PBL) of the Earth. These measurements therefore provide significant information to upscale from site to region. Moreover, the number of CO_2_ concentration measurements above the land surface, made by either tower or aircraft, is steadily increasing. Data are collected by numerous agencies around the world, for instance, the National Oceanic and Atmospheric Administration's (NOAA's) Earth System Research Laboratory (ESRL) monitors CO_2_ in the atmosphere as a contribution to the North American Carbon Program (NACP) [82]. In addition, Peters *et al.* [78] suggest that direct satellite observations of CO_2_ are available already for the upper troposphere, whereas near-surface CO_2_ from space will become available within several years to augment the current efforts.

Previous efforts to interpret the signal of regional CO_2_ exchange making use of tower concentration data have focused on simple one-dimensional PBL budgets that rely on gradients in CO_2_ concentrations between the PBL and the free troposphere [77,79,83-85]. These methods are limited to monthly resolution because of the need to smooth and average over several synoptic events [86].

The atmosphere integrates surface fluxes over many temporal and spatial scales and links scalar sources and sinks with concentrations and fluxes. This principle has been successfully used to develop inverse models to estimate annual carbon budgets [87-90]. However, due to model limitations and paucity of continental CO_2_ observations these studies have yielded carbon fluxes only at coarse resolution, over large spatial regions (i.e., at continental scale, [91]). Recently, Stephens [89] and Yang [90] showed that a large set of atmospheric inverse model results were inconsistent with free troposphere CO_2_ concentration. Transport biases are one of the largest unknown sources of error in flux inversions [88].

A powerful way to use all these CO_2_ data is in a data assimilation system, which combines diverse data and models into a unified description of a physical/biogeochemical system consistent with observations [13]. Such a new data assimilation system called CarbonTracker, has been built in the NOAA ESRL, using a state-of-the-art atmospheric transport model coupled to an ensemble Kalman filter [78]. The first release of CarbonTracker marks a significant step in our ability to monitor month-by-month surface sources and sinks of CO_2_ [78], with all results, data and code of CarbonTracker freely available from NOAA ERSL's CarbonTracker web site, http://carbontracker.noaa.gov.

### 3.3. Stable C Isotope Measurements

The information on the biological and physical processes that exchange CO_2_ between terrestrial ecosystems and the atmosphere is recorded by the signals of ^13^C /^12^C ratio in the atmosphere CO_2_. The stable isotope ratio of CO_2_ (δ^13^C) in the atmosphere contains unique information to study the overall balance of surface CO_2_ fluxes [92,93]. As reviewed by Suits *et al.* [94], C isotopes can be helpful in investigations of the following four aspects at ecosystem and local scales: (i) plant water-use efficiency and the response of plants to changes in precipitation and relative humidity [95-100], (ii) variation in light distribution and stand structure [101-104], (iii) recycling of respired CO_2_ [105-109], and (iv) determining the relative contributions of photosynthesis and respiration to the total net ecosystem exchange [110-114].

Multiple efforts to measure stable C isotopes at both flux towers and flask stations around the world have been achieved. Several new techniques (e.g., automated measurement systems, tunable diode laser (TDL) spectrometer and pulsed quantum cascade laser spectrometer) have also been applied to this investigation [115-117], isotope measurements, however, are still lacking considering the land surface diversity/heterogeneity. This shortage of long-term measurements and of sampling frequency still limits isotopic studies and applications to various spatial/temporal scales.

Efforts to expand CarbonTracker to assimilate observations of C isotopes (^13^CO_2_, ^14^CO_2_) and other observation types (eddy-flux measurements, satellite radiances) are underway. Such observations could facilitate attribution of carbon fluxes to specific processes such as fossil fuel burning, biomass burning, or agricultural food and biofuel production [78].

### 3.4. Satellite Monitoring

Satellite-borne remote sensing offers unique opportunities to parameterize land surface characteristics over large spatial extents at variable spatial and temporal resolutions. For example, the Moderate Resolution Imaging Spectroradiometer (MODIS) provides a global dataset every 1–2 days with 36 bands. The spatial resolution of MODIS (pixel size at nadir) is 250 m for channels 1 and 2 (0.6–0.9 μm), 500 m for channels 3 to 7 (0.4–2.1 μm) and 1,000 m for channels 8 to 36 (0.4–14.4 μm), respectively. Data from the satellite-borne MODIS are currently used in the calculation of global weekly GPP at 1 km spatial resolution [10]. Other sensors, the Landsat Thematic Mapper sensors carried onboard the Landsat series of satellites, acquire images at a 30 m spatial resolution with a 16 day interval.

In general, water evapotranspired from ecosystems into the atmosphere will reduce the land surface temperature (*T_a_*). Reduction in soil moisture will decrease plant transpiration and evaporation from soil and plant surfaces. Reduction in ET will increase *T_a_. T_a_* can be derived from remotely-sensed thermal-infrared (TIR) band (8-14 microns) from various operational satellites. Based on the relationship between *T_a_* and ET, remotely sensed *T_a_* has been used to estimate regional ET [118-121]. The existing thermal imaging sensors provide adequate coverage of thermal dynamics that are useful for operational monitoring applications of ET. For example, thermal images at 15 minutes intervals and at a spatial resolution of 5 kilometers can be obtained from the NOAA Geostationary Operational Environmental Satellites (GOES), and TIR data at a fine spatial resolution (60 m or 120 m) with a much longer time interval (16 days) have been provided by the Thematic Mapper (TM) and ETM+ instruments on Landsat 5 and Landsat 7.

ET, the largest component of water loss from ecosystems, plays an important role in affecting soil moisture, vegetation productivity, C cycle, and water budgets in terrestrial ecosystems [122-124]. Verstraeten *et al.* [12] provided a comprehensive review of remote sensing methods for assessing ET and soil moisture content across different scales and Kalma *et al.* [11] reviewed satellite-based algorithms for estimating ET and land surface temperatures at local, regional and continental scales, with particular emphasis on studies published since the early 1990s.

In addition, as Marquis and Tans [13] reviewed, satellite-based instruments can also provide information about CO_2_ concentration in the atmosphere, but no current satellite-borne instrument comes close to providing the accuracy, precision, and continuity required to determine regional CO_2_ concentrations and local fluxes. Future satellites, including the Greenhouse Gases Observing Satellite (GOSAT) [125], are expected to provide more accurate CO_2_ measurements than do today's satellites [13,126].

### 3.5. Other Airborne Measurements

Besides satellite monitoring, other airborne observation techniques (e.g., aircraft, airplane and land surface remote sensing) have been developed rapidly since the latest decade. For instance, a new approach is LiDAR (Light Detection and Ranging), which is a remote sensing technology that determines distances to an object or surface using laser pulses. LiDAR data have proved to be highly effective for the determination of three dimensional forest attributes. The suitability of airborne LiDAR for the determination of forest stand attributes including leaf area index (LAI) and the probability of canopy gaps within different layers of canopy has been widely acknowledged by various studies [127,128]. The interpreted LiDAR data have been further used for landscape C modeling and scaling [129,130].

## 4. Modeling of C and Water Dynamics in Terrestrial Ecosystems

The land surface of the Earth represents significant sources, sinks, and reservoirs of C, heat and moisture to the atmosphere. C and energy fluxes and water cycles at soil-atmosphere and plant-atmosphere interfaces are therefore important land surface processes. Due to the complexity and non-linearity of C, N and water dynamics in terrestrial ecosystems, various modeling tools are needed for better understanding of these biogeochemical and hydrological processes and their feedback mechanisms with the land surface climate system [131]. It is well known that realistic simulations of C, N and water dynamics in terrestrial ecosystems is of critical importance, not only for the surface microclimate, but also for the large-scale physics of the atmosphere [132-134]. Such models can be flagged by land surface, ecosystem and hydrological models based on their objectives and emphases. The former focus on ecosystem processes and the interactions between ecosystems and the atmosphere; while the latter place emphasis on the land surface hydrology processes, including lateral flow resulting from catchment topography.

### 4.1. Land Surface and Ecosystem Modeling

Global climate and the global carbon cycle are controlled by exchanges of water, carbon, and energy between the terrestrial biosphere and atmosphere. Thus land surface models (LSMs) are essential for the purpose of developing predictive capability for the Earth's climate on all time scales [135]. Most current LSMs can be associated with three broad types [136]: soil-vegetation-atmosphere transfer schemes (SVATS), potential vegetation models (PVMs), and terrestrial biogeochemistry models (TBMs).

The first generation of SVATS evolved from simple bucket schemes focusing on soil water availability [137], through the schemes of Deardorff [138]. Marked improvements of the second generation (e.g., BATS [136], SiB [139,140], and CLASS [141,142]) from the first generation are the separation of vegetation from soil and the inclusion of multiple soil layers for dynamic heat and moisture-flow simulations [143]. The second generation SVATS firstly modeled plant physiology in an explicit manner in GCMs (General Circulation Model or Global Climate Model) [144]. For most second-generation SVATS, land cover was fixed, with seasonally-varying prescriptions of parameters such as reflectance, leaf area index or rooting depth [145-148]. Some SVATS incorporated satellite data to characterize more realistically the seasonal dynamics in vegetation function [146,149]. The latest (third generation) SVATS used more recent theories relating photosynthesis and plant water relations to provide a consistent description of energy exchange, ET, and C exchange by plants [143,150]. In our effort in understanding the impact of climate change on terrestrial ecosystems, energy, water, and C cycles need to be modelled simultaneously [150,151]. Recently, most of SVATS have thus been enhanced to include the CO_2_ flux between the land surface and the atmosphere, such as SiB2 [150], IBIS [152], NCAR-LSM [153], BATS [134], CLASS-C [154] and EASS [143].

The earlier generation of PVMs comprised a suite of schemes that focus on modeling distributions of vegetation as a function of climate [155,156] without influences of anthropogenic or natural disturbance. The second generation of PVMs included more sophisticated modules to account for factors controlling vegetation distributions, such as competition, varying combinations of plant functional types, and physiological and ecological constraints [157].

TBMs developed from scaling up local ecological models, are process-based models that simulate dynamics of energy, water, and C and N exchange among biospheric pools and the atmosphere [136]. Few of the existing TBMs incorporate PVMs. These models are not applicable to transient climate change experiments without coupling with PVMs.

In recent decades, the interactions among soil, vegetation and climate have been studied intensively and modeled successfully on the basis of water and energy transfer in the soil-vegetation-atmosphere system [136,140,142,158]. Also the construction and refinement of LSMs have received increasing attention [150,159,160]. Combination of these three different LSMs and utilization of remotely sensed land surface parameters are critical in the future LSM development, because of (1) the tight coupling of exchanges of water, energy and C between the land surface and the atmosphere; (2) the sophisticated impact/feedback mechanisms between climate change and terrestrial ecosystems; and (3) increasingly strong anthropogenic alterations to land cover. On-line coupling of a LSM with a GCM is needed for studying interannual to multi-decadal climate variations.

Several model intercomparisons have focused on evaluating SVATS and TBMs with particular objectives. For instance, the Project for Intercomparison of Land-surface Parameterization Schemes (PILPS) was initiated to evaluate an array of LSMs existing in GCMs [144]; while the AMMA (African Monsoon Multidisciplinary Analysis) Land Surface Model Intercomparison Project (ALMIP) is being conducted to get a better understanding of the role of soil moisture in land surface processes in West Africa [161]. Coordinated land surface modeling activities have improved our understanding of land surface processes [161].

### 4.2. Spatially-distributed Hydrological Processes Modeling

Hydrology and ecosystem have, for the most part, been studied independently. Most LSMs and ecosystem models make an assumption of “flat Earth” with the absence of lateral redistribution of soil moisture. On the other hand, hydrological models have mostly been concerned with runoff production. Spatially-distributed models are needed, especially for hydrological simulation objective, because of heterogeneity of land surface and non-linearity of hydrological processes. Spatially-distributed hydrological models are not only able to account for spatial variability of hydrological processes, but enable computation of internal fluxes and state variables. Such kinds of models are increasingly applied to simulate spatial variability of forcing variables (e.g., precipitation), physiographic characteristics, detailed processes and internal fluxes within a catchment [162-167].

### 4.3. Modeling Dynamics of Stable C Isotopic Exchange between Ecosystem and the Atmosphere

It is recognized that the atmospheric measurements are still too sparse, relative to its spatial variability, to be used for inferring the surface flux at high spatial resolution [168]. The use of the isotope ratio as an additional constraint to identify various C sources and sinks can contribute to a significant reduction in the uncertainty. Though available isotopic datasets are being accumulated quickly [117,169-171], isotope measurements are still lacking considering land surface diversity and heterogeneity. This shortage of long-term measurements and of sampling frequency still limits C isotopic studies.

Mechanistic ecosystem models that couple micrometeorological and eco-physiological theories have the potential to shed light on how to extend efforts and applications of stable isotopes of CO_2_ to global C budgeting, because biophysical models have the capacities of simulating isotope discrimination in response to environmental perturbations and can produce information on its diurnal, seasonal and interannual dynamics. Few biophysical models, however, have been developed to assess stable C discrimination between a plant canopy and the atmosphere [94,87,135,]. Most existing biophysical models are based on individual leaf level discrimination equations given by Farquhar *et al.* [173,174] and only focus on the land surface layer (ignoring vertical and horizontal advection effects beyond 50∼100 m above the ground [172]. However, in nature, the convective boundary layer (CBL) integrates the effects of photosynthesis, respiration, and turbulent transport of CO_2_ over the landscape [107,175]. The influence of the CBL cannot be ignored when using isotope composition of CO_2_ to investigate biological processes [176], because the effect of atmospheric stability on turbulent mixing/diffusion has an important impact on scalar fluxes and concentration fields within and above canopies [177,178]. Few such models considering the CBL effects on isotope fractionation have been developed to date [107,178-182].

### 4.4. Modeling Coupled C, N and Water Dynamics—An Ecohydrological Approach

C and N dynamics and hydrological processes are closely linked. The stomatal conductance (*g*_s_) is the key linkage between C assimilation (photosynthesis) and transpiration. An empirical equation is used in the second-generation LSMs to calculate *g*_s_, which is hypothesized to be controlled by the environmental conditions [31]. While field and laboratory studies have documented that leaf photosynthesis also affects *g*_s_. Therefore, Ball *et al.* [24] proposed a semi-empirical stomatal conductance formulation (Ball-Woodrow-Berry model), in which *g*_s_ is controlled by both photosynthesis and the environmental conditions. Most of third-generation LSMs (Ecological models, e.g., SiB2 [139,150]; CN-CLASS [28]; *Ecosys* [183-184; and EASS [143]) fully couple photosynthesis and transpiration processes by employing the Ball-Woodrow-Berry stamatal conductance formulation.

In addition to the coupling of hydrological condition and C assimilation through the linkage of *g*_s_, C assimilation is also coupling with N dynamics through another biochemical parameter, $V_{c\max}^{25}$ — maximum carboxylation rate at 25 °C. In the photosynthesis model proposed by Farquhar *et al.*[174], the net photosynthetic rate *A_net_* at leaf level is a function of two tightly-correlated parameters $V_{c\max}^{25}$ and $J_{c\max}^{25}$ (the maximum electron transport rate at 25 °C), and is calculated as:

(1) $$A_{\text{net}}=\min(A_{c},A_{j})-R_{d}$$

where *A_c_* and *A_j_* are Rubiso-limited and light-limited gross photosynthesis rates, respectively, and *R_d_* is the daytime leaf dark respiration and computed as *R_d_* = 0.015 *V_c max_. A_c_* and *A_j_* are expressed as:

(2a) $$A_{c}=V_{c\max}\frac{C_{c}-\Gamma^{*}}{C_{c}+K_{c}(1+O_{c}/K_{o})}$$

and:

(2b) $$A_{j}=J_{\max}\frac{C_{c}-\Gamma^{*}}{4(C_{c}+2\Gamma^{*})}$$

where *C_c_* and *O_c_* are the intercellular CO_2_ and O_2_ mole fractions (mol mol^−1^), respectively; Γ* is the CO_2_ compensation point without dark respiration (mol mol^−1^); *K_c_* and *K_o_* are Michaelis-Menten constants for CO_2_ and O_2_ (mol mol^−1^), respectively. In the nutrient-limited stands, *A_net_* is generally limited by *A_c_*, while *A_c_* is dominantly controlled by a parameter *V_c_* _max_ (see Eqn. 2a). Many research results showed $V_{c\max}^{25}$ is very sensitive to leaf N status (more specifically leaf Rubisco-N) [134,185-187]. As a result in some ecosystem models (i.e., C&N-CLASS [23]), $V_{c\max}^{25}$ is calculated as a nonlinear function of Rubisco-N following observations made by Warren and Adams [134]:

(3) Vcmax25(N)=α[1−exp(−1·8Nr0]

where α is the maximum value of $V_{c\max}^{25}$ and *N_r0_* is the leaf Rubisco-N (g N m^−2^ leaf area) in the top canopy.

The coupled C, N and water processes have been carefully considered in most of the third-generation LSMs (e.g., SiB2 [139,140,150]); CN-CLASS [28] and *Ecosys* [183,184]), the models' grids, however, are isolated from their neighboring grids mainly due to the availability of input data. Vertical soil hydrological processes are hard to be realistically simulated if the lateral flows are ignored by assuming that the Earth is “flat”. However, simulations of the topographically-driven lateral water flows are important components in most of spatially-distributed models, while the detailed ecophsiological processes are weakly represented [188]. Much effort to bridge these two different models has been increasingly made [33,35,188-194]. However, a model coupling approach—a full combination of ecosystem model and hydrological model, i.e., ecohydrological modeling, is still lacking.

### 4.5. Applications of Remotely-sensed Data in Ecohydrological Modeling

Remote sensing techniques, which inherently have the ability to provide spatially comprehensive and temporally repeatable information of the land surface, may be the only feasible way to obtaining data needed for land surface and ecological modeling [140,194-197]. The most common rationale for interfacing remote sensing and land surface-ecosystem models is using remotely sensed data as model inputs [198]. These input data, corresponding to forcing functions or state variables in ecological modeling, include LC, LAI, normalized difference vegetation index (NDVI), and the fraction of photosynthetically active radiation (*f_PAR_*) [140,199-201]. Another effort is the direct estimation of GPP and NPP [202,203] of biomass [203-204] and of plant growth [205,206], by making use of *f_PAR_* and NDVI. It has been shown that the direct estimation has lower accuracy than the integration of remotely sensed data with process based models [202].

Remote sensing data have also been used to parameterize hydrological models [194,195,207]. For instance, a hydrological model (TerrainLab) was further developed using remote sensing as inputs [194]. TerrainLab is a spatially distributed, process-oriented hydrological model using the explicit routing scheme of Wigmosta *et al.* [208]. This model has been applied to flat areas (such as boreal and wet land region, [188,195,209]), but it has not yet been applied to mountainous areas.

Different from traditional hydrological models, which have coarse spatial resolutions, the grid-based-distributed ecohydrological models have a high demand for spatial data [195,210]. Some researchers highlight that the main obstacles in current distributed ecohydrological modeling is the lack of sufficient spatially distributed data for input and model validation [211]. Remote sensing can potentially fill in some of the gaps in data availability and produce means of spatial calibration and validation of distributed hydrological models. As a result the application of remote sensing techniques in hydrological studies and water resources management has progressed in the past decades (see review by [195]).

In general, the applications of remotely sensed data in ecohydrological modeling can be in the two ways [194,195,207,210-218]: (i) multispectral remote sensing data are used to quantify surface parameters, such as vegetation types and density. Although the usefulness of remote sensing data is widely recognized, there remain few cases where remote sensing data have been actually used in ecohydrological simulations. Difficulties still exist in choosing the most suitable spectral data for studying hydrological processes as well as in interpreting such data to extract useful information [194,195,219]; and (ii) processed remote sensing data are used to provide fields of hydrological parameters for calibration and validation of ecohydrological models, such as precipitation [195,220], and soil moisture [221-224]. Koster *et al.* [224] pointed out that remote sensing data take the form of emitted and reflected radiances and thus are not the type of data traditionally used to run and calibrate models. Hence, it is important to understand and develop relationships between the electromagnetic signals and hydrological parameters of interest [194]. Kite and Pietroniro [195] stated that the use of remote sensing in hydrological modeling was limited. Even though a number of new sensors have been launched since then and research has documented that remote sensing data have promising perspectives, operational uses of satellite data in hydrological modeling still appear to be in its infancy [211].

## 5. Research gaps in C and Water Flux Estimates and Scaling Approaches

**Figure 3.**
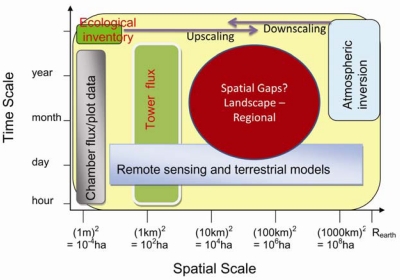
Temporal and spatial scales of different approaches.

A variety of methods are being used in the C and water cycles studies. As shown in Figure 3, different approaches have different temporal and spatial scales. The most direct measurements of the terrestrial C flux are made either at the plot scale (10^−2^–10^1^ m^2^), e.g., using biometric methods and various forms of chamber, or at the ecosystem (patch) scale (10^4^–10^6^ m^2^), using the EC technique. Ecohydrological / ecosystem modeling and remote sensing estimations are generally available across variable spatiotemporal scales. These estimates are normally available within a nested framework that permits a progressive comparison of measurements made by surface instrumentation (scale: 1 to 10 m), surface flux equipment (10 m to 1 km), airborne remote sensing equipment (100 m to several km), satellite remote sensing (30 m to global scale) and EC tower (1–3 km).

The atmosphere integrates surface fluxes over many temporal and spatial scales and links scalar sources and sinks with concentrations and fluxes. This principle has been successfully used to develop inverse models to estimate annual C budgets [225-230]. However, due to model limitations and paucity of continental CO_2_ observations these studies have yielded C fluxes only at coarse resolution, over large spatial regions [231-233].

Progress in C balance studies has been achieved at both ends of the spatial scale spectrum, either large continents (larger than 10^6^ km^2^, e.g., global inverse modeling) or small vegetation stands (less than 1–3 km^2^, e.g., EC-measurements). Methods to estimate CO_2_ sources and sinks at the intermediate scale (i.e., landscape to regional scales) between continental and local scales are less well advanced. Moreover, the C cycle in different regions can vary markedly in response to changing climate [5]. Reliable estimates of terrestrial C sources and sinks at landscape to regional spatial scales (finer than those used in global inversions and larger than local EC flux measurements and roughly defined as the range between 10^2^ and 10^6^ km^2^) are required to quantitatively account for the large spatial variability in sources and sinks in the near-field of a measurement location [234], as well as fundamental to improving our understanding of the C cycle [235].

It is generally considered unreliable to upscale stand-level fluxes (i.e., EC measurements) to a region by simple spatial extrapolation and interpolation because of the heterogeneity of the land surface and the nonlinearity inherent in ecophysiological processes [236]. It is also challenging to apply atmospheric inversion technique to regional scales for quantifying annual C budgets because at such intermediate scales the atmosphere is often poorly constrained [86,237]. Moreover, aggregation errors and errors in atmospheric transport, both within the PBL and between the PBL and free troposphere, can also be obstacles to using these approaches to obtain quantitative estimates of regional C fluxes [76]. Hence, there is a strong motivation to develop methods to quantify and validate estimates of the C balance at these intermediate scales [76-77,79,86]. Observations of CO_2_ over the continent within the PBL reflect exchange processes occurring at the surface at a regional scale (10^2^–10^5^ km^2^). The flux information contained in CO_2_ concentration data represents footprints of up to 10^5^ km^2^ [75,76], which are several orders of magnitude larger than the direct EC-flux footprint. This information is therefore needed in our effort to upscale from site to region. Moreover, the number of CO_2_ mixing ratio measurements above the land surface, made by either tower or aircraft, is steadily increasing. Previous efforts to interpret the signal of regional CO_2_ exchange making use of tower concentration data have focused on simple one-dimensional PBL budgets that rely on gradients in CO_2_ concentrations between the PBL and the free troposphere [79,84]. These methods are limited to monthly resolution because of the need to smooth and average over several synoptic events [86].

## 6. Future Research Directions

A synthetic research framework is needed to strength the less well researched areas as reviewed in Section 5: bottom-up and top-down approaches integrating scalable (footprint and ecosystem) models and a spatially nested hierarchy of observations which include multispectral remote sensing, inventories, existing regional clusters of eddy-covariance flux towers and CO_2_ mixing ratio towers and chambers.

The current research trends and the future directions in this field include: (i) A synthesis aggregation method—integrating ecohydrological and isotopic models, remote sensing and component flux data, is becoming a pragmatic approach towards a better understanding of the coupled C, N and water dynamics at landscape/watershed scales; and (ii) The landscape- and regional-scale C fluxes are being estimated using an integrated approach involving direct land surface measurements, remote sensing measurements, and ecosystem-, footprint- and inversion- modeling.

### 6.1. Development of a Spatially Explicit Ecohydrological Modeling Framework

Coupled modeling will help refine the experimental and instrumental design and generate cross-disciplinary hypotheses that can be tested in the experiment. Ecohydrological models are powerful tools for quantitative and predictive understanding the coupled C, N and water mechanisms. Spatially-explicit ecohydrological modeling can be used to infer aspects of the land surface system that are difficult to measure by mass and energy balance, and will be critical to improving the accuracy of forecasts of landscape change and C dynamics in the real world. While developing a process-based, coupled-system model is a significant task, the coupling to existing models may provide a relatively easy way forward. For example, a spatially explicit, process-based ecohydrological model, EASS-TerrainLab, is developed to improve the representation of the coupled C, N and hydrological processes by integrating of two existing models (an ecosystem LSM model—EASS and a distributed hydrological model—TerrainLab).

#### 6.1.1. Reviewing of the Existing EASS Model [112]

**Figure 4.**
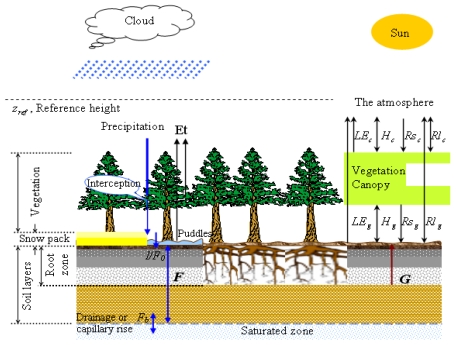
Structure of the EASS model. Three components (soil, vegetation and the atmosphere) are considered in EASS, which are integrated with two interfaces. The right panel illustrated energy fluxes between these three components. *LE, H, Rs, Rl*, and *G* are the latent heat flux, sensible heat flux, shortwave radiation, longwave radiation, and soil conductive heat flux, respectively; the subscripts *g* and *c* present the energy fluxes at soil-canopy and canopy-atmosphere interfaces, respectively. The left panel describes soil water fluxes. The symbol *F* represents conductive water flux between soil layers, and *F_0_* represents the incoming water flux from the surface to the top soil layer (i.e., the actual infiltration rate *I*), and *F_b_* is the water exchange (drainage or capillary rise) between the bottom soil layer and the underground water [143].

EASS is based on a single layer vegetation canopy overlying a seven-layer soil, and includes physically-based treatment of energy and moisture fluxes from the vegetation canopy and through it. It also incorporates explicit thermal separation of the vegetation from the underlying ground [134]. Moreover, EASS includes a scheme with stratification of sunlit and shaded leaves to avoid shortcomings of the “big leaf” assumption [197,238]. It has been referred as a “two-leaf” canopy model [81,239]. The structure of EASS is shown in Figure 4.

With spatially explicit input data on vegetation, meteorology and soil, EASS can be run pixel by pixel over a defined domain, such as Canada's landmass, or any of its parts, or the globe. EASS has flexible spatial and temporal resolutions, as long as the input data of each pixel are defined.

In short, EASS has the following characteristics: (i) satellite data are used to describe the spatial and temporal information on vegetation [143]; (ii) energy and water exchanges and carbon assimilation in the soil-vegetation-atmosphere system are fully coupled and are simulated simultaneously. The Ball-Woodrow-Berry stamatal conductance formulation [24] was employed (see Section 4.4); (iii) the energy and C assimilation fluxes are calculated with stratification of sunlit and shaded leaves to avoid shortcomings of the “big-leaf” assumption; and (iv) the lateral movement of water is ignored.

#### 6.1.2. Reviewing of the TerrainLab Model [157]

TerrainLab [194] was adopted concepts from Wigmosta *et al.* [208] and it has been tested in the Southern Study Area (SSA) of the BOREAS region to study the spatio-temporal variation of ET and soil moisture [143]. The horizontal boundary of the simulated area is a watershed delineated from a digital elevation model (DEM) where divides between neighboring watersheds are identified. Vertically, the simulation extends from the saturated zone in the soil to the top of vegetation canopy. Within a watershed, the forest ecosystem is divided into basic spatial units, or pixels in remote sensing. Each pixel is treated as a unique vegetation-soil system, except for the ground and runoff water exchanges. Basic model simulations of the physical and biological processes are made at the pixel scale. According to the need of simulating hydrological processes, a pixel is vertically divided into five strata, i.e., overstory, understory, litter or moss layer, soil unsaturated zone, and soil saturated zone. Precipitation, solar radiation, topographic parameters, land cover, LAI, and soil properties are the major inputs to the model.

The soil water balance component of TerrainLab allows for the spatially explicit simulation of topographically driven lateral subsurface flow and its influence on water table depth and soil water content dynamics based on a raster grid DEM [194]. TerrainLab uses the explicit routing scheme of Wigmosta *et al.* [208]. In this approach the soil profile is subdivided into an unsaturated and a saturated zone. Saturated hydraulic conductivity is assumed to be depth-dependent, while other hydraulic parameters are considered to be vertically homogenous. However, the assumption of vertical homogeneity of permanent wilting point, field capacity, and porosity represents a major simplification in peat lands. The lateral movement of water between each raster grid cell (pixel) of the modeling domain and its maximum eight neighboring pixels based on a multi-predilection-flow algorithm occurs in the saturated zone, i.e., as groundwater flow. Groundwater follows the local hydraulic gradient (3 × 3 pixels) that is assumed to be approximated by local ground surface slopes [194,208].

#### 6.1.3. Development of an Ecohydrological Model by Coupling of EASS with TerrainLab

The coupled model (EASS-TerrainLab) explicitly describes ecohydrological processes (coupled C, N and water dynamics). Each process will be explicitly calculated separately for overstory and understory with sunlit and shaded leaf groups. Vertical hydrological processing will be adequately described using detailed water balance equations, the topographically driven lateral water flows will be considered for both saturated and unsaturated soil layers, and their influence on soil water content and water table depth are calculated based on a raster grid DEM [194] using the explicit routing scheme of Wigmosta *et al.* [208].

#### 6.1.4. Model Calibration and Validation for EASS-TerrainLab

Model calibration and validation will conducted in three ways: (i) Modeled surface runoff will be compared with the watershed discharge measurements; (ii) Simulated soil water storage and its changes will be verified with several soil water measurement profiles; and (iii) Simulated ET will be validated with EC-flux tower measured ET based on footprint averaging and the remotely sensed spatially-distributed ET.

#### 6.1.5. Model Sensitivity Analysis and Runs under Different Scenarios

A set of sensitivity analysis will conducted to test the coupled capacity. The model will be run under several different scenarios to explore: (i) the effects of surface and sub-surface base flow on GPP and ET; (ii) the influence of mesoscale topography on hydrological processes (runoff and soil storage) and C exchange (water use efficiency and light use efficiency); (iii) the influence of photosynthesis on ET by comparing simulation results using different stomatal conductance schemes; and (iv) the effects of representations of spatial variability on simulations of photosynthesis and ET.

### 6.2. Landscape and Regional C and Water Fluxes Estimation: An Upscaling Framework

**Figure 5.**
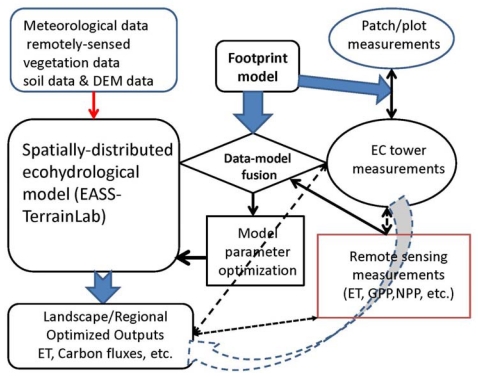
An upscaling framework synthetically integrating ecohydrologcal and footprint modeling, remote sensing and land surface measurements.

Figure 5 schematically shows a synthetic upscaling framework for estimating landscape and regional C and water budgets with a reasonable accuracy. Using a data-model fusion with footprint weighting method to optimize the ecohydrological model's parameters is strongly suggested because the land surface is normally heterogeneous. This is a key component in the upscaling framework. Model parameters are optimized by minimizing the ‘cost’ function [240]:

(4) $$J(\text{x})=\frac{1}{2}\left[{\left(O-Y(\text{x})\right)}^{\text{T}}C_{o}^{-1}(O-Y(\text{x})+{\left(\text{x}-x_{\text{b}}\right)}^{T}{\text{P}}_{\text{b}}^{-1}\left(x-x_{\text{b}}\right)\right]$$

where **x** is the vector of unknown parameters and **x_b_** is a vector of *a priori* values of **x**; ***O*** is the vector of observations and **Y** is the nonlinear model results, **C_o_** is the covariance matrix of observations and ***P*_b_** is the covariance matrix of *a priori* parameters. Different from general data-fusion method, **Y** is weighting averaged by footprint *f_j_* for each pixel (*j*) within the footprint domain as **Y** = ∑**Y***_j_f_j_*.

We also note that the dynamics of the PBL is tightly coupled with the land surface processes (e.g., energy / water / C fluxes). But for simplification, we proposed off-line modeling instead of the on-line coupling with regional climate model or the GCMs. The meteorological driving inputs for the ecohydrological model, therefore, are required for model runs. These inputs can either acquire from spatially extrapolation from climate station (including EC flux towers) measurements, or from the re-analysis data (e.g., the data provided by National Centers for Environmental Prediction (NCEP)), or from the regional climate modeling outputs.

## 7. Summary

After comprehensive reviewing of a variety of approaches being used in research on the C/water cycles, the concluding remarks are summed the following:

Research gaps in this field are (i) The coupled terrestrial C, N and hydrological dynamics are far from well understood, especially at landscape (watershed) and regional scales; (2) Much progresses have been achieved at the extreme ends of the spatial-scale spectrum, either large regions/continents or small vegetation stands. Because of the heterogeneity of the land surface and the nonlinearity inherent in ecophysiological and ecohydrological processes in response to their driving forces, it is difficult to upscale stand level results to regions and the globe by extrapolation. Budgets of carbon and water at landscape intermediate regional scales (10^2^–10^5^ km^2^) have large uncertainties.

A coupled spatially-explicit ecohydrological model is a powerful tool for quantitative and predictive understanding of the coupled C, N and water mechanism. This modeling framework can be used to infer aspects of the land surface system that are difficult to measure, and will be critical to improving the accuracy of forecasts of landscape change and C dynamics in the real world.

Combining and mutually constraining the bottom-up and top-down methods to reduce their uncertainties using data assimilation techniques is a practical and effective means to derive regional C and water fluxes with reasonably high accuracy. In the proposed upscaling framework by this paper, spatially nested hierarchy of observations, including multispectral remote sensing, inventories, existing regional clusters of EC flux towers and CO_2_ mixing ratio towers and chambers, are able to integrated using scalable (footprint and ecosystem and ecohydrological) models and data-model fusion techniques.
